# Functionalization of Bacterial Cellulose Nonwoven by Poly(fluorophenol) to Improve Its Hydrophobicity and Durability

**DOI:** 10.3389/fbioe.2019.00332

**Published:** 2019-11-15

**Authors:** Ji Eun Song, Carla Silva, Artur M. Cavaco-Paulo, Hye Rim Kim

**Affiliations:** ^1^Human Convergence Technology Group, Korea Institute of Industrial Technology, Ansan, South Korea; ^2^Centre of Biological Engineering, University of Minho, Braga, Portugal; ^3^Department of Clothing and Textiles, Sookmyung Women's University, Seoul, South Korea

**Keywords:** bacterial cellulose (BC), fluorophenol, polymerization, laccase, hydrophobization, functionalization

## Abstract

The present study aims to improve the hydrophobicity and durability of bacterial cellulose (BC) nonwoven by functionalization with poly(fluorophenol). To this end, laccase was first entrapped onto BC and then used to polymerize the fluorophenol {4-[4-(trifluoromethyl) phenoxy] phenol} *in-situ*. The polymerization of fluorophenol by laccase was confirmed through ^1^H NMR and MALDI-TOF analyses. The effect of poly(fluorophenol) on BC nonwoven was determined by evaluation of the surface hydrophobicity and olephobicity properties such as water contact angle (WCA), oil contact angle (OCA), surface energy and water/oil absorption time. After BC functionalization with poly(fluorophenol) (20 mM), the WCA increased from 54.5 ± 1.2° to 120 ± 1.5° while the surface energy decreased (11.58 ± 1.4 mN/m). The OCA was also increased from 46.5 ± 2.5° to 87 ± 2° along to the decrease of surface energy (8.7 ± 1.5°). X-ray photoelectron spectroscopy (XPS) analysis confirmed an increase in the fluorine content in BC from 5.27 to 17.57%. The findings confirmed the polymerization of fluorophenol by laccase and its entrapment onto a BC nanofiber structure. The durability of the functionalization with poly(fluorophenol) was confirmed by evaluating the washing fastness, tensile strength after washing and dimensional stability. The results indicate that the functionalized BC nonwoven had higher tensile strength (×10 times) and better dimensional stability (30%) than the non-functionalized BC nonwoven material.

## Introduction

Bacterial cellulose (BC) is composed of nanofibrilar structure that efficiently self-assemble and form a nanofibre network of 10–100 nm in diameter. This material possess high purity, high degree of polymerization, crystallinity, and high water holding capacity (Lee, 2011; Ashjaran et al., 2012; Llanos, 2012; Yim et al., 2017; Fernanda et al., 2017; Song et al., 2017; Nam and Lee, 2019). Specifically, BC is made of a fibrous three dimensional structure (e.g., nonwoven) from which fabric can be obtained without knitting or weaving. BC nonwoven is characterized by high density of inter-and intra fibrillar hydrogen bonds that offer a large surface area, good mechanical properties and high hydrophilicity (Kalia et al., 2014; Kucinska-Lipka et al., 2015; Chan et al., 2018; García and Prieto, 2019). However, there are several drawbacks of the hydrophilicity of BC nonwoven: this property, along with high moisture uptake, can cause dehydration and poor durability. When BC nonwoven is exposed to moist or wet conditions, it loses its original shape, making it difficult to recover its original shape and strength. In order to improve BC nonwoven durability, it is imperative to control and improve its specific properties such as hydrophobicity (Andresen et al., 2006; Tomé et al., 2011; Fijałkowski et al., 2015). Thus, we explored a method for improving the hydrophobicity, and consequently, the durability of BC nonwoven.

Several previous studies have proposed strategies for improving the physical and chemical properties of plant cellulose fibers such as flax, coconut fiber (Herrero Acero et al., 2014), and jute fiber (Wu et al., 2016) using the laccase-mediated polymerization of functional polymer, namely fluorophenol. The fluorine-containing monomer fluorophenol{4-[4-(trifluoromethyl) phenoxy] phenol} has demonstrated hydrophobic properties to lignocellulose fibers through laccase-mediated polymerization (Marie et al., 2004; Kudanga et al., 2010a; Sun et al., 2013; Herrero Acero et al., 2014; Saravanakumar et al., 2016; Slagman et al., 2016; Wu et al., 2016). However, BC nonwoven has not been subjected to improve its properties using laccase-mediated fluorophenol polymer.

Therefore, in this study, BC nonwoven was subjected to functionalize by laccase-mediated polymerization of fluorophenol. For this purpose, laccase was first entrapped inside BC swelled nanofiber structure. Thereafter, the fluorophenol was polymerized *in-situ* by laccase. Poly(fluorophenol) produced by laccase-mediated polymerization was chemically identified. The main goal of this study was to confirm the effect of functionalization with poly(fluorophenol) on the hydrophobic and durability of BC nonwoven. To this end, the hydrophobic and olephobic performance of BC nonwoven were evaluated by water and oil contact angle, surface energy, and water/oil absorption time. Improved durability of BC nonwoven was confirmed by evaluating the washing fastness, tensile strength, and dimensional stability after washing.

## Materials and Methods

### Materials and Equipment

Glucose and peptone were purchased from Merck Co. Ltd. (Germany). The yeast extract, analytical grade sodium hydroxide hydrate pellets, sodium chloride, sododecyl benzenesulfonate (C_18_H_29_NaO_3_S), sodium acetate (C_2_H_3_NaO_2_), acetic acid (CH_3_COOH), and fluorophenol {4-[4-(trifluoromethyl) phenoxy] phenol} were purchased from Sigma Chemical Co. (St. Louis, Mo, U.S.A.). Analytical grade glacial acetic acid was purchased from Fisher Chemical (Fair Lawn, N.J, U.S.A.). Commercial laccase (Novozym 51003) (EC 1.10.3.2) from *Thermobifida fusca* was obtained from Novozymes (Bagsveard, Denmark). The static cultivation of BC was conducted in an incubator (SI-600R, JEIO TECH Co., Daejeon, Korea). The swelling of the BC was performed using an ultrasonic bath (UC-20, JEIO TECH Co., Daejeon, Korea). A freeze dryer (Operon Co. Ltd., Kimpo, Korea) and an oven dryer (OF-22G, JEIO TECH Co., Daejeon, Korea) were used to dry the BC. The laccase-mediated polymerization of fluorophenol and washing fastness tests were conducted in a shaking water bath (BS-31, Jeio Tech, Korea). The WCA, OCA and surface energy were determined using the contact angle measurement system (DSA100, KRÜSS Inc., Germany). The absorbance was monitored through UV-visible spectrophotometry (Multimode Microplate Reader Synergy™ Mx and Gen5™, BioTek, Instruments, Winooski, VT, U.S.A.). The morphology of BC samples was examined using a scanning electron microscope (SEM, JSM-7600F, JEOL Korea Ltd., Japan). The tensile strength was evaluated using a tensile testing machine (Digital Tensility Strength, ASA-211-1, Seoul, Korea). The X-ray photoelectron spectroscopy (XPS) was analyzed using a PHI 5000 VersaProbe (ULVAC PHI, Japan). Polymerization of fluorophenol was characterized by ^1^H NMR spectroscopy (Bruker Avance III 400, Bremen, Germany) at 400 MHz and Matrix-Assisted Laser Desorption/Ionization with time-of-flight (MALDI-TOF, Bruker Daltonics Gmbh, Bremen, Germany).

### Preparation of BC Nonwoven

BC was cultured using a method previously reported by Han et al. (2018). BC nonwoven samples used for the subsequently experiments had similar size (2 × 2 cm), with an average thickness of 0.25 ± 0.05 mm and average weight of 0.5 ± 0.02 g after drying.

**Figure 1:**
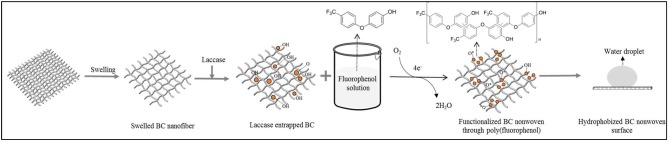
Schematic illustration of functionalization BC nonwoven through laccase-mediated polymerization of fluorophenol aiming to improve the hydrophobicity of BC nonwoven.

As shown in Figure 1, BC was first swelled as described by Song et al. (2018). After swelling, laccase was entrapped into swelled BC nanofiber by adding 20% (W/V) of laccase (245.5 U/ml) to 2 ± 0.8 g of BC in a 10 ml acetate buffer (pH 5, 0.1 M) for 30 min. To assume the amount of entrapped laccase in BC, laccase activity was measured spectrophotometrically by monitoring the enzymatic oxidation rate of 2,20-azinobis(3-ethylbenzothiazoline-6-sulphonic acid) (ABTS) to its cation radical (ABTS^+^) at 420 nm (ε_420_ = 36,000 M^−1^ cm^−1^) in 0.1 M acetate buffer (pH 4.0) at 25°C. One unit (U) of activity is defined as the amount of enzyme forming 1 μmol/min of ABTS^+^. All spectrophotometric measurements were carried out by UV-visible spectrophotometry.

Several concentrations of fluorophenol solution (5, 10, and 20 mM) were prepared by dissolving in a 1:2 (ethanol: acetate buffer-pH 5, 0.1 M solution) (Wu et al., 2016). BC samples containing entrapped laccase were immersed in the fluorophenol solutions at 50°C for 24 h, and under shaking at 135 rpm. After 24 h, the solutions were collected and BC samples were washed using an aqueous solution of 1% sodium dodecyl sulfate (SDS) for 2 h to remove the monomers and oligomers unabsorbed onto BC (Figure 1; hereinafter, it was referred to as functionalized BC nonwoven). The control BC samples were prepared without addition of laccase.

### ^1^H NMR and MALDI-TOF

The polymerization of fluorophenol by laccase was confirmed through ^1^H NMR and MALDI-TOF spectrometry. Dimetyl sulfoxide (DMSO) was used as suitable solvent to dissolve the powders after freeze drying. ^1^H NMR spectra were acquired on a Bruker DRX-500, AVANCE 400, and Varian Inova 750 spectrometers at 25°C (1H: 499.82 MHz). Chemical shifts (DMSO) are reported near 2.5 ppm. Mnova software 9.0 (Mestrelab Research) was used to analyze all the spectra (Pilz et al., 2003). MALDI-TOF analysis of the new oligomers was conducted using 2,5-dihydroxy benzoic acid (DHB) as the matrix (≥99.5%). The mass spectra were acquired on an Ultra-flex MALDI-TOF mass spectrophotometer equipped with a 337 nm nitrogen laser. The samples were dissolved in a TA30 (30% acetonitrile/70% TFA) solution and mixed with a 20 mg/mL solution of DHB (1:1) or with a saturated solution of α-Cyano-4-hydroxycinnamic acid (CHCA). Then a volume of 2 μl was placed in the ground steel plate (Bruker part no. 209519) until dry. The mass spectra were acquired and analyzed using in linear positive mode.

### Surface Properties of BC Nonwoven Functionalized With Poly(Fluorophenol)

#### Water Contact Angle (WCA), Oil Contact Angle (OCA), Surface Energy, and Water/Oil Absorption Time

The effect of poly(fluorphenol) on the surface properties of BC nonwoven was determined by water contact angle (WCA), oil contact angle (OCA), surface energy and water/oil absorption time (Dong et al., 2014). The WCA/OCA, surface energy were determined using the water contact angle measurement system. A dosing volume of water/oil droplet of 3 μl was placed in the samples using a Hamilton 500 μl syringe at a suitable distance to the testing platform. Each measurement was performed on a different spot of the sample and the data presented is the average of at least three measurements. The apparatus was used together with specialized software for determination of surface energy. In addition, the Girifalco–Good–Fowkes–Young model was used to calculate the average surface energy. The water absorption time was recorded as the time taken by the specular reflectance of a droplet to completely disappear. Each sample was tested in at least three spots and the data used is the average of all measurements (Dong et al., 2014). Methylene blue (C.I. 52015) was dissolved in distilled water, after which a 5 μl drop of this solution was dripped onto the surface of the functionalized BC nonwoven, and the absorption behavior was observed.

#### XPS Analysis

The changes in the surface chemistry of functionalized BC nonwoven were determined through XPS using a PHI 5000 VersaProbe instrument equipped with a monochromator AI Ka source (1486.6 eV). A narrow scan was completed at pass energy of 40 eV, followed by obtaining high-resolution scans of C1s (284.6 eV). For quantitative analysis, a subtraction method in the linear line background was applied to all the main spectral bands and the respective areas were calculated. These data and the respective X-ray cross-sectional values were used to calculate the percentage atomic concentration of each element. The component analysis of the spectral regions was performed through peak fitting using pure Gaussian line shapes.

#### Scanning Electron Microscopy (SEM)

The surface morphology of BC nonwoven samples was examined using a scanning electron microscope (SEM) at 5,000 magnifications (SEM, JSM-7600F, JEOL Korea Ltd., Japan).

### Evaluation of the Durability of Functionalized BC Nonwoven

The durability properties of the functionalized BC nonwoven were evaluated by measurement their washing fastness, tensile strength and dimensional stability, respectively. The washing fastness was evaluated by measuring the change of WCA over washing time. The functionalized BC nonwoven samples were washed in distilled water for 180 min at 25°C in a water bath (110 rpm). In addition, the change of WCA was evaluated before and after washing. The tensile strength was also evaluated before and after washing. For evaluating the tensile strength, BC nonwoven samples were washed in distilled water for 5 min, and then dried for 3 h at 25°C. After drying, the samples were subjected to a tensile test using a load cell of 100 N at a crosshead speed of 0.17 mm/s. The results were based on the average of at least three measurements using a tensile testing machine, in accordance with the ISO 13934-1:2013 method.

The dimensional stability was evaluated using the ISO 7771:2012 method. BC nonwoven (70 × 250 mm) samples were marked at four locations for measuring the dimensional change (Song et al., 2019). Thereafter, the samples were immersed in distilled water that contained a wetting agent (sodium dodecyl benzene sulfonate, C_18_H_29_NaO_3_S), for 60, 120, and 180 min. After immersion, BC nonwoven samples were dried in an oven dryer (OF-22G; JEIO TECH Co.) at 35°C for 5 h. The samples were then evaluated and their dimensional stability (%) was calculated according to the following Equation (1):

(1) $$\text{Dimensional stability }(\%)=\frac{{\text{A}}_{\text{mm}}}{{\text{B}}_{\text{mm}}}\;\times 100$$

where B_mm_ is the measurement between marks before immersion and A_mm_ is the measurement between marks after immersion.

## Results and Discussion

### Fluorophenol Polymerization by Laccase

#### ^1^H NMR Analysis

**Figure 2:**
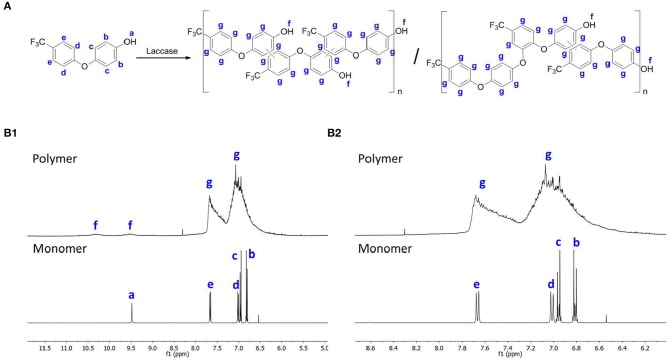
**(A)** Proposed representation of the reactional scheme for the polymerization of fluorophenol; **(B1)** ^1^H NMR spectra of the polymer and monomer of fluorophenol **(B2)** expansion of the ^1^H NMR spectra of **(B1)** to δH 6.0 at 8.7 ppm.

The ^1^H NMR spectra evidences a more complex pattern for the polymerized samples than the monomer (Figure 2B1). As shown in Figures 2B1, 2B2, the fluorophenol monomer peaks were observed between δ_H_ 6.8 and 9.5 ppm. The OH group (proton a) appears at δ_H_ 9.5 ppm as a singlet, whereas all other protons of the monomer were observed as doublets between δ_H_ 6.8 and 7.4 ppm (protons b, c, d, and e) (Wu et al., 2016). The spectrum of the final products indicates a mixture of peaks in the aromatic zone, which confirms a mixture of oligomers/polymers (Wu et al., 2016). Based on the differences between the signal intensities of the OH and the aromatic peaks, we predict that the reaction occurred through carbon-carbon linkage or oxygen-carbon linkage, as shown in Figure 2A. This assumption is based on the differences between the signal intensities with the OH signal and aromatic peaks. The different reaction possibilities between monomers lead to a complex mixture, as evidenced by NMR spectroscopy which in turns hard to predicted the occurrence of only one structure. Despite the heterogeneity observed, this technique allowed us to confirm the fluorophenol polymerization by laccase.

#### MALDI-TOF Analysis

**Figure 3:**
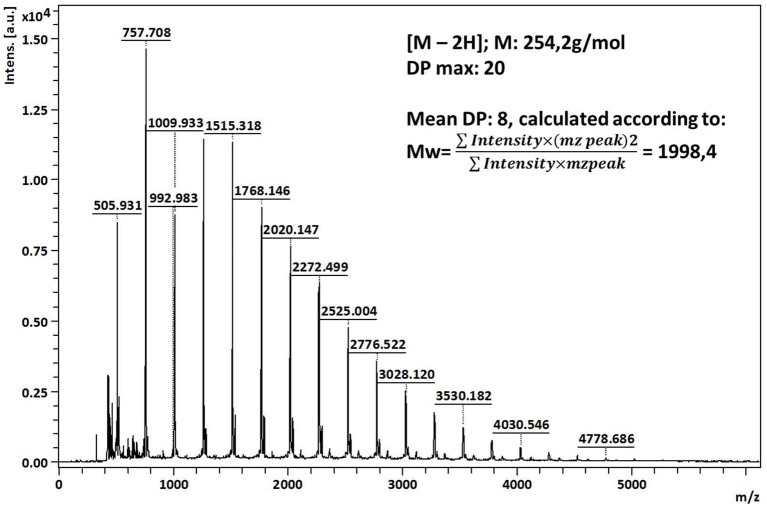
MALDI-TOF MS of fluorophenol polymers produced by laccase-mediated polymerization.

The final products of polymerization were also analyzed by MALDI-TOF spectrometry in order to determine the degree of polymerization. The mass spectrum of poly(fluorophenol) show peaks from 505 to 4,778 m/z with the M_w_ calculated 1998.4 and the M_n_ 1566.1 (PDI = 1.28) (Figure 3). The maximum m/z detected correspond to a DP_max_ = 20 and a DP_avg_ = 8, inferred by MALDI data, and by considering that the m/z difference between each peak (252 m/z) correspond to the monomer unit of fluorophenol (C_13_H_9_F_3_O_2_). The polydispersity value (>1) reveals some heterogeneity of the analyzed samples, which may be related to the different oligomeric species that were obtained, along with the ionization events that occurred during the analysis (Marie et al., 2004; Kudanga et al., 2010a).

### Surface Properties of BC Nonwoven Functionalized With Poly(Fluorophenol)

#### Water Contact Angle (WCA), Surface Energy, and Time of Water Absorption

Fluorophenol was polymerized by entrapped laccase (190 U/mL) in the BC nanofiber structure. The fluorine molecules were converted into hydrophobic groups, which were then entrapped into the BC nonwoven structure (Marie et al., 2004; Kudanga et al., 2010b; Wu et al., 2016). As indicated in Table 1A, BC nonwoven demonstrated a low water contact angle (54.5 ± 1.2°) with high surface energy (51.12 ± 0.8 mN/m). After BC nonwoven was treated by only laccase (Table 1B), the water contact angle was slightly increased (72.5 ± 1.5°). On the other hand, the BC nonwoven samples functionalized through fluorophenol in the absence of laccase (Table 1C) display an increase in the water contact angle. The greatest increase in WCA was observed for samples that were functionalized with poly(fluorophenol) and obtained through laccase-mediated polymerization (112.2 ± 3°) (Table 1D). This increase in the WCA was accompanied by a remarkable decrease in the surface energy (14.26 ± 1.8 mN/m). These findings confirm the polymerization of fluorine monomers as well as the consequent functionalization of the BC nonwoven samples with the poly(fluorophenol) produced. Altogether the time of water absorption increased to values considered for hydrophobic samples (>10 min). These data might be explained by the covering of the BC samples, mainly composed by hydroxyl groups at the surface along with the poly(fluorophenol). Due to its hydrophobic nature caused by the fluorine groups in its composition, the polymer confers hydrophobicity to the functionalized samples. The significant decrease in the surface energy values is directly related with the bonding between the hydrocarbon chain of poly(fluorophenol) and the BC network structure, which makes it more difficult for the fibers to absorb water molecules (Wu et al., 2016).

**Table 1:** WCA, surface energy, and water absorption time of **(A)** BC nonwoven, **(B)** BC nonwoven + laccase, **(C)** BC nonwoven + fluorophenol, and **(D)** BC nonwoven + fluorophenol + laccase [10 mM fluorophenol; laccase (190 U/mL); 50°C for 24 h].

	**WCA** ** (^**°**^)**	**Surface energy** ** (mN/m)**	**Water absorption time**
**(A)** BC nonwoven	54.5 ± 1.2	51.12 ± 0.8	2 min 40 ± 5 s
**(B)** BC nonwoven + laccase	72.5 ± 1.5	33.8 ± 1.2	1 min 55 ± 5 s
**(C)** BC nonwoven + fluorophenol	88.5 ± 2	27.9 ± 1.4	5 min 45 ± 8 s
**(D)** BC nonwoven + fluorophenol + laccase	112.2 ± 3	14.3 ± 1.8	>10 min

The effect of the amount of poly(fluorophenol) on the surface properties of BC nonwoven was evaluated by varying the concentration of the initial monomer (5–20 mM of fluorophenol). As indicated in Table 2, the WCA of the functionalized BC nonwoven increased in proportion with the initial fluorophenol concentration. The highest value of WCA (120 ± 1.5°) and the lowest surface energy (11.58 ± 1.4 mN/m) were observed for samples incubated with 20 mM of fluorophenol. These results conform with the previous studies of Wu et al. (2016), which reported that concentrations of monomers up to 20 mM are expected to provide good polymerization yield and good surface coverage of hydrophobic groups such as fluorine, leading to high hydrophobicity of BC. However, at higher monomer concentrations of up to 20 mM, the polymerization yield decreased when the incremental tendency of the contact angle began to weaken and reached the maximum value. This is explained by the CF_2_ on the fiber surface being nearly saturated; therefore, increasing the deposition of fluorophenol concentration did not have a significant impact on the wettability of the fiber surface (Song et al., 2018).

**Table 2:** Water contact angle (WCA) and surface energy of BC nonwoven samples functionalized with poly(fluorophenol) and obtained through a laccase-mediated polymerization [5–20 mM fluorophenol; laccase (190 U/mL); 50°C for 24 h].

		**WCA** ** (^**°**^)**	**Surface energy** ** (mN/m)**
Fluorophenol concentration (mM)	5	104.7 ± 1.3	22.4 ± 0.5
	10	112.2 ± 2.0	14.26 ± 1.2
	20	120 ± 1.5	11.58 ± 1.4

#### Oil Contact Angle (OCA), Surface Energy, and Time of Water Absorption

The oil contact angle, surface energy, and oil absorption time of BC nonwoven samples also were evaluated. As indicated in Table 3A, BC nonwoven showed the lowest olephobicity (46.5 ± 2.5°) that is similar with BC nonwoven treated by only laccase (48.7 ± 0.5°) (Table 3B). This can be assumed to be because the fluorine monomer did not polymerize and was desorbed from the BC surface. In contrast, the high OCA of 87.2 ± 2° was observed at functionalized BC nonwoven with poly(fluorophenol) (Table 3D), resulting from the high fluorine contents on BC nonwoven (**Table 5C**).

**Table 3:** OCA, surface energy, and oil absorption time of **(A)** BC nonwoven, **(B)** BC nonwoven + laccase, **(C)** BC nonwoven + fluorophenol, and **(D)** BC nonwoven + fluorophenol + laccase [10 mM fluorophenol; laccase (190 U/mL); 50°C for 24 h].

	**OCA** ** (^**°**^)**	**Surface energy** ** (mN/m)**	**Oil absorption time**
**(A)** BC nonwoven	46.5 ± 2.5	37.17 ± 2.5	1 min 45 ± 10 s
**(B)** BC nonwoven + laccase	48.7 ± 0.5	35 ± 2.8	6 min 10 ± 5 s
**(C)** BC nonwoven + fluorophenol	66.7 ± 1.2	28.12 ± 0.7	>10 min
**(D)** BC nonwoven + fluorophenol + laccase	87.2 ± 2	8.7 ± 1.5	>10 min

Table 4 shows the hydrophobic behavior of the BC nonwoven samples determined by deposition of a water solution containing methylene blue dye. It was observed that the dye droplets on the surface of the BC nonwoven samples functionalized with poly(fluorophenol) (>60 min) were easily absorbed by the non-functionalized BC samples (<10 min). This result confirms the efficient polymerization of flurophenol by laccase and the role of the newly formed polymer in conferring a hydrophobic character to BC nonwoven surfaces.

**Table 4:** Absorption profiles of a water droplet with a disperse dye (C.I. Disperse blue 284) solution on **(A)** BC nonwoven, **(B)** BC nonwoven + fluorophenol, and **(C)** BC nonwoven + fluorophenol + laccase [10 mM fluorophenol; laccase (190 U/mL); 50°C for 24 h].

**Time (min)**	**0**	**5**	**10**	**30**	**60**
**(A)** BC nonwoven	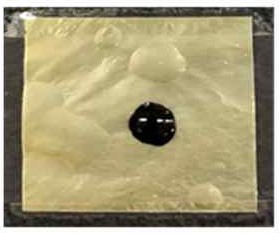	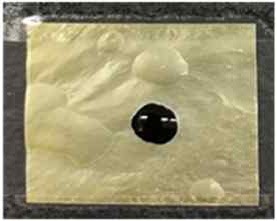	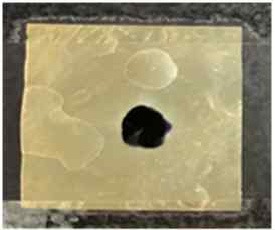	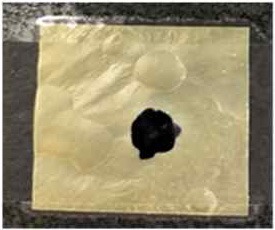	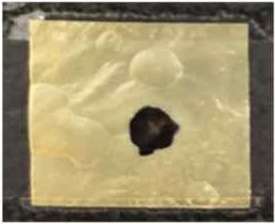
**(B)** BC nonwoven + fluorophenol	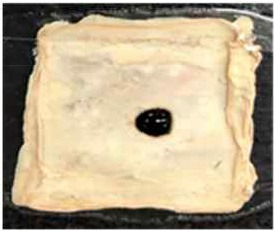	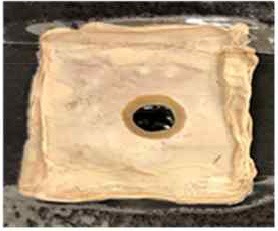	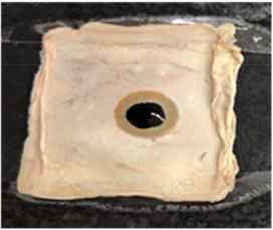	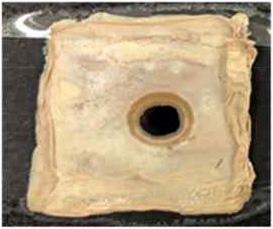	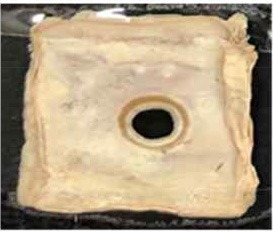
**(C)** BC nonwoven + fluorophenol + Laccase	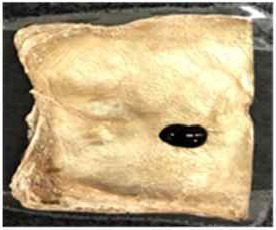	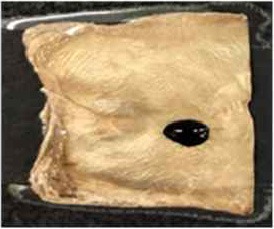	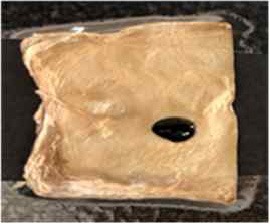	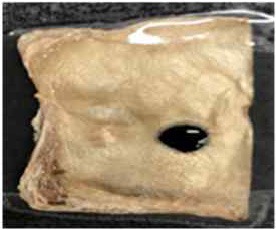	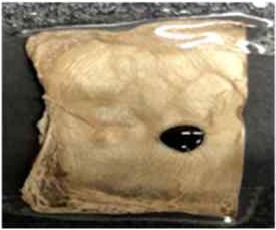

#### XPS Analysis

**Figure 4:**
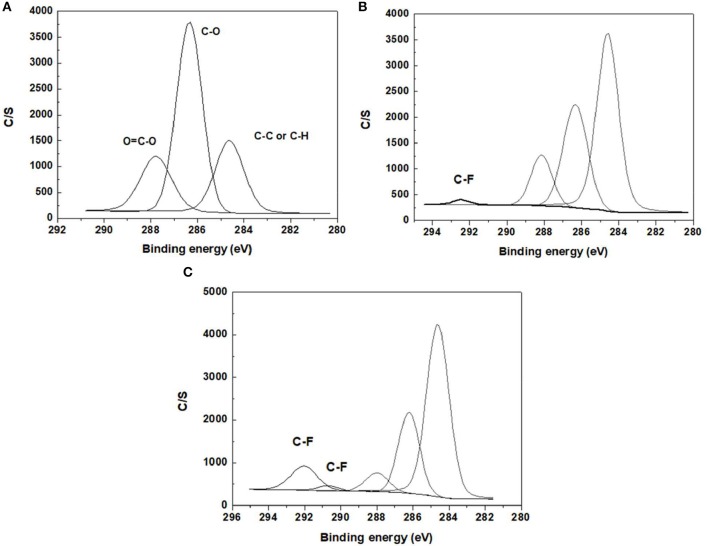
The x-ray photoelectron spectra of **(A)** BC nonwoven, **(B)** BC nonwoven + fluorophenol **(C)** BC nonwoven + fluorophenol + laccase [20 mM fluorophenol; laccase (190 U/mL); 50°C for 24 h].

XPS analysis was used to study the element content of the functionalized BC nonwoven samples; its results are shown in Figure 4 and Table 4. The binding energy of the quantified C1s signal can be divided into different energy peaks. As shown in Figures 4A–C, the characteristic peaks of BC such as C-C or C-H (284.51 eV), C-O (286.08 eV), and O = C-O (288.16 eV) peaks appeared (Pertile et al., 2010). Table 5 indicates the change in the atomic composition of BC surface. Non-functionalized BC (Table 5A) is mainly composed of C and O, whereas in the functionalized BC nonwoven, the peak of CF_2_ appeared at 292.6 eV (Figure 4B and Table 5B). The change was remarkable for the functionalized BC nonwoven due to the presence of polymerized fluorophenol (Figure 4C and Table 5C). The additional peak corresponding to CF_2_ appeared at 290.5 eV, along with an increase in the fluorine content from 5.27 to 17.57 %. The fluorine atoms present in the matrix may improve the hydrophobic interaction with the fluorinated oligomers during sample target preparation (Marie et al., 2004; Wu et al., 2016). The increase in F is due to insertion of the hydrocarbonate radical from fluorophenol (Herrero Acero et al., 2014), which confirms the polymerization of fluorophenol by entrapped laccase.

**Table 5:** The atomic composition (%) of **(A)** BC nonwoven, **(B)** BC nonwoven + fluorophenol, **(C)** BC nonwoven + fluorophenol + laccase [20 mM fluorophenol; laccase (190 U/mL); 50°C for 24 h].

	**Surface atomic composition (%)**				
	**C1**	**N**	**O**	**F**	**Na**
**(A)** BC nonwoven	61.07	0.78	36.60	0.70	0.84
**(B)** BC nonwoven + fluorophenol	61.50	0.37	25.81	5.27	7.06
**(C)** BC nonwoven + fluorophenol + laccase	63.49	0.84	15.31	17.57	2.79

#### SEM Analysis

**Figure 5:**
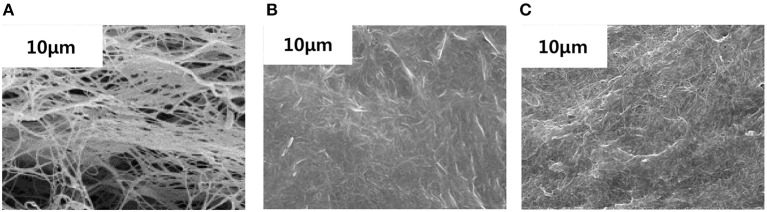
The SEM micrographs of **(A)** BC nonwoven, **(B)** BC nonwoven + fluorophenol, **(C)** BC nonwoven + fluorophenol + laccase [20 mM fluorophenol; laccase (190 U/mL); 50°C for 24 h].

As shown in Figure 5A, BC nonwoven show a flat surface with a fibrilar network structure consisting of BC nanofiber ribbons. BC nanofibrils can be observed at the surface of the multilayered structure, evidenced by a well-organized three-dimensional network (Lin et al., 2013). The surface of BC nonwoven functionalized with the monomer fluorophenol in the absence of laccase (Figure 5B) and elucidated the effect of laccase. As indicated by Figure 5B, BC surface is not regularly coated, which displays a typical BC nanofiber structure. Only random agglomerates of fluorophenol were observed between the BC nanofibers. On the contrary, BC nonwoven functionalized with poly(fluorophenol) and catalyzed by laccase showed remarkable changes in terms of morphology (Figure 5C). After functionalization, the polymer is dispersed along the BC network, resulting in film-like samples (Figure 5C). The hydrogen bond interactions between the hydroxyl groups of BC nanofibers and the polymer are the main causes of this effect (Battirola et al., 2018).

### Durability of Functionalized BC Nonwoven With Poly(Fluorophenol)

**Figure 6:**
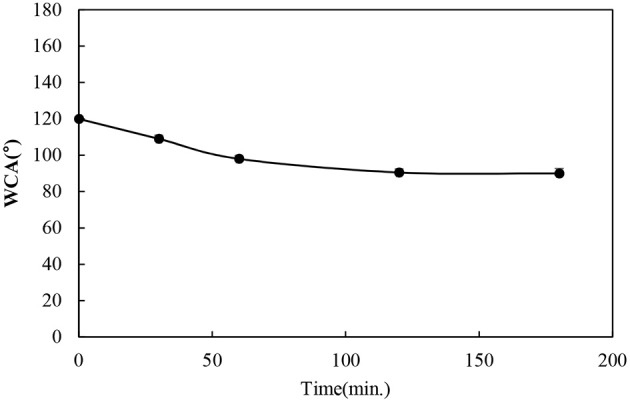
Water contact angle (WCA) of functionalized BC nonwoven after washing.

The durability of the functionalized BC nonwoven samples was evaluated by measuring the washing fastness, tensile strength, and dimensional stability studies, respectively. To confirm the durability of functionalized BC nonwoven against the washing, it was repeatedly washed for 180 min. As shown in Figure 6, after washing for 180 min, although the WCA decreased, it remained higher than 90°. The good fixing capacity of poly(fluorophenol) is explained by the formation of ether linkages between the hydrocarbon chains of the fluorophenol polymer and the OH groups of BC (Bashar et al., 2017).

**Figure 7:**
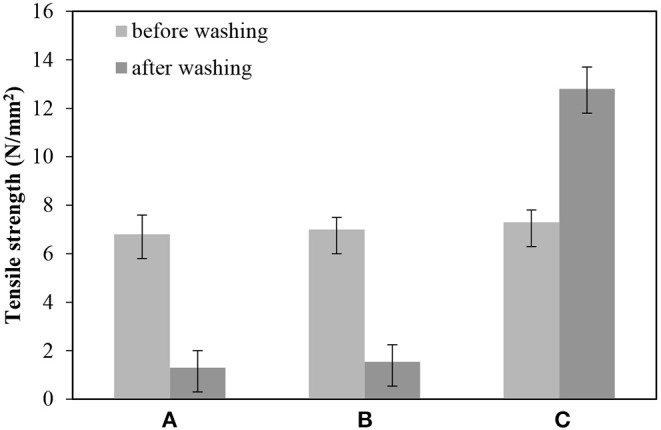
The tensile strength (N/mm^2^) of **(A)** BC nonwoven, **(B)** BC nonwoven + fluorophenol, **(C)** BC nonwoven + fluorophenol + laccase [20 mM fluorophenol; laccase (190 U/mL); 50°C for 24 h].

The tensile strength of BC samples was evaluated before and after washing. As shown in Figure 7A, the tensile strength of BC nonwoven decreased from 6.8 ± 0.5 to 1.8 ± 0.7 N/mm^2^ after washing, indicating a loss of ~70% of the original tensile strength. Since original BC has poor resistance to water absorption and high water uptake, BC loss its original strength when exposed to water (Wan et al., 2009). As shown in Figure 7B, the tensile strength of BC nonwoven treated by fluorophenol alone was not remarkably improved. On the other hand, BC nonwoven functionalized with poly(fluorophenol) demonstrated an increase of ~7.3 ± 0.5 N/mm^2^ (Figure 7C) in the tensile strength after washing. The 3-fold increase of this property confirm the deposition of polymerized fluorophenol onto BC surface; this along with the removal of the adsorbed water may have caused an increase in the interactions between BC fibers. These results comply with those of previous studies. Herrero Acero et al. (2014) reported that coconut and flax fibers exhibited enhanced mechanical properties when functionalized with poly(phenols) through laccase-assisted reactions (Herrero Acero et al., 2014). Thus, the hydrophobic molecules formed through adherence with the fibers conferring improved mechanical properties. Similarly, Kudanga et al. (2010b) reported that the functionalization of BC with a hydrophobic substrate polymerized by laccase lead to an increase in the material's strength in wet conditions (Kudanga et al., 2010b).

**Figure 8:**
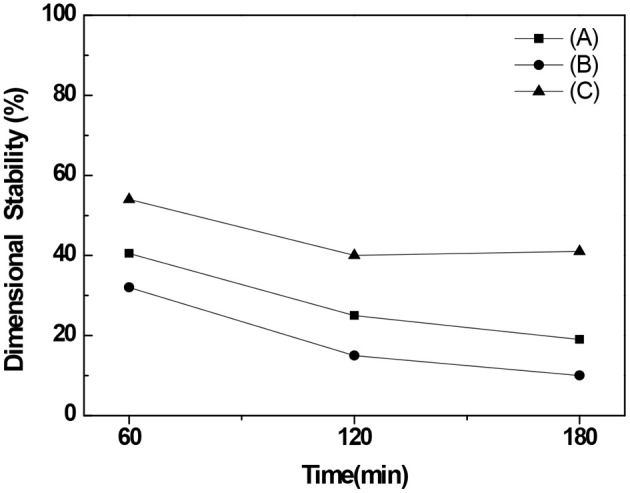
The dimensional stability (%) of **(A)** BC nonwoven, **(B)** BC nonwoven + fluorophenol, **(C)** BC nonwoven + fluorophenol + laccase [20 mM fluorophenol; laccase (190 U/mL); 50°C for 24 h].

Examining the dimensional stability revealed that its values for BC nonwoven declined from 32 to 10% after immersion for 180 min in water containing a detergent for 180 min (Figure 8A). Comparing with the BC nonwoven functionalized with fluorophenol (Figure 8B), the BC nonwoven functionalized with poly(fluorophenol) (Figure 8C) retained over 45% of their initial dimensions, even after a prolonged immersion time. The dimensional stability of the functionalized samples was governed by their chemical compositions. The strong adhesion between BC nanofibers and polymerized fluorophenol promoted a decrease in water absorption, further preventing structure deformation. The overall findings indicate an improvement in the durability of the BC samples after functionalization with poly(fluorophenol).

## Conclusion

This study aimed to improve the hydrophobicity and durability of BC nonwoven through functionalization with poly(fluorophenol). Laccase was efficiently entrapped in BC structure and used as a catalyst for *in situ* polymerization with fluorophenol, as confirmed by ^1^H NMR and MALDI-TOF data. The *in situ* polymerization of BC nonwoven samples with poly(fluorophenol) resulted in hydrophobic surfaces. The water contact angle increased from 54.5 ± 1.2° to 120 ± 1.5° along with a decrease in the surface energy (11.58 ± 1.4 mN/m). In addition, the oil contact angle was improved from 46.5 ± 2.5° to 87.2 ± 2°. The chemical composition after functionalization was confirmed by XPS, which revealed an increase of the fluorine content from 5.29 to 17.57%. The new polymer was uniformly dispersed on the BC structure, as evidenced by SEM analysis. Considering the ultimate goal of this study, there was a significant improvement in the durability properties such as washing fastness, tensile strength after washing, and dimensional stability, respectively. These findings demonstrates that functionalized BC possess the potential as durable nonwoven textile material for technical clothing, shoes, bags, and others, which have been unexplored thus far. Moreover, depending on the application of BC nonwoven, more durability may be required. To this end, additional functionalization conditions suitable for BC nonwoven should be investigated in future studies.

## Data Availability Statement

All datasets generated for this study are included in the article/supplementary material.

## Author Contributions

JS was responsible for experimental details, writing and paper revision. CS was responsible for experimental details and writing details. AC-P was responsible for experimental details. HK was the supervisor of the work.

### Conflict of Interest

The authors declare that the research was conducted in the absence of any commercial or financial relationships that could be construed as a potential conflict of interest.
